# Prevalence and Risk Factors of Asymptomatic Bacteriuria Among Pregnant Women Attending a Tertiary Care Hospital in Jaipur, India

**DOI:** 10.7759/cureus.111585

**Published:** 2026-06-26

**Authors:** Simran Saini, Jitendra Panda, Shiv Prakash Sharma, Nilofar Khayyam, Varun Kumar Saini

**Affiliations:** 1 Department of Microbiology, Rajasthan University of Health Sciences College of Medical Sciences (RUHS-CMS), Jaipur, IND; 2 Department of Preventive Medicine, Rajasthan University of Health Sciences College of Medical Sciences (RUHS-CMS), Jaipur, IND; 3 Department of Anesthesiology, Rajasthan University of Health Sciences College of Medical Sciences (RUHS-CMS), Jaipur, IND

**Keywords:** antenatal screening, asymptomatic bacteriuria, pregnancy, primigravida, urinary tract infection

## Abstract

Introduction: Asymptomatic bacteriuria (ASB) in pregnancy increases the risk of pyelonephritis, preterm labor, and low birth weight. However, routine screening is not uniformly practiced in India. This study aimed to determine the prevalence of ASB and identify demographic and obstetric risk factors among pregnant women attending a tertiary care hospital in Jaipur, Rajasthan.

Methods: A laboratory‑based prospective observational study was conducted from May 2025 to September 2025. Clean‑catch midstream urine samples were collected from 310 pregnant women. Significant bacteriuria was defined as ≥10⁵ colony‑forming units/mL. Clinical and demographic data were analyzed using the chi‑square test; p < 0.05 was considered significant.

Results: Of 310 pregnant women, 90 (29.0%) were culture‑positive. Among culture‑positive women, 65 (72.2%) were asymptomatic. The highest culture positivity was seen in the third trimester (50/147, 34.0%) and in women aged 25-29 years (36/117, 30.8%). Primigravida women accounted for 43/90 (47.8%) of positive cases. Statistically significant associations with culture positivity were found for parity (χ² = 7.192, p = 0.027), gravida (χ² = 12.458, p = 0.002), and age group (χ² = 4.884, p = 0.027).

Conclusions: ASB affects nearly three‑quarters of pregnant women with urinary tract infection in this tertiary care setting. The highest risk is observed in primigravida women and those in the third trimester. Routine urine culture screening for all pregnant women, irrespective of symptoms, is essential to prevent maternal and fetal complications.

## Introduction

Urinary tract infection (UTI) is the second most common infection in the community and the leading cause of morbidity among pregnant women [1]. During pregnancy, anatomical and hormonal changes, including progesterone‑induced ureteral dilatation, mechanical compression by the gravid uterus, and increased glycosuria, create an environment that favors bacterial colonization [2,3].

Asymptomatic bacteriuria (ASB) is defined as the presence of ≥10⁵ colony-forming units (CFU)/mL of a single uropathogen in a properly collected urine specimen from a person without symptoms of UTI [4]. In pregnancy, ASB is clinically significant because up to 30% of untreated cases progress to acute pyelonephritis, which is associated with preterm labor, low birth weight, and increased perinatal mortality [5,6]. Other adverse outcomes include maternal anemia, intrauterine growth retardation, preterm premature rupture of membranes, and postpartum endometritis [7].

The reported prevalence of ASB in pregnant women in India ranges from 3% to 24% [8,9]. However, routine urine culture screening is not consistently performed in many antenatal clinics, especially at primary and secondary health centers [10]. Most studies from India have focused on symptomatic UTI, leaving a gap in understanding the true burden of ASB and its associated risk factors.

Therefore, this study was planned to determine the prevalence of ASB among pregnant women attending a tertiary care hospital in Jaipur and to identify demographic and obstetric factors associated with culture‑positive UTI.

## Materials and methods

Study design and setting

A laboratory‑based prospective observational study was conducted from May 2025 to September 2025 at the Department of Microbiology, Rajasthan University of Health Sciences College of Medical Sciences, Jaipur, in collaboration with the Department of Obstetrics and Gynecology. The study was approved by the Institutional Ethics Committee (approval letter no. RUHS‑CMS/Ethics Comm./2025/441).

Study population

Pregnant women attending the antenatal clinic were enrolled after written informed consent was obtained. The inclusion and exclusion criteria used for participant selection are presented in Table 1.

**Table 1:** Inclusion and exclusion criteria Participants meeting any one of the exclusion criteria were excluded from the study

Inclusion criteria	Exclusion criteria
Pregnant women aged ≥18 years	Antibiotic use within the previous 72 hours
Any trimester of pregnancy	Indwelling urinary catheter or recent catheterization, and structural urinary tract abnormalities
Willingness to participate in the study	Known chronic kidney disease, diabetes mellitus, known HIV infection/AIDS, and immunosuppressive therapy
Written informed consent provided	Preeclampsia, multiple gestation, and high-risk pregnancy

Sample collection and processing

Clean‑catch midstream urine specimens (10-20 mL) were collected in sterile, wide‑necked, leak‑proof containers. Perineal cleansing with clean water and sterile gauze was performed before collection. Samples were transported to the microbiology laboratory within two hours. If immediate transport was not possible, samples were refrigerated at 4°C-6°C.

Each sample was inoculated onto Blood agar, MacConkey agar, and cystine-lactose-electrolyte-deficient agar using a calibrated loop (1 µL) and incubated aerobically at 37°C for 18-24 hours. Significant bacteriuria was defined as ≥10⁵ CFU/mL of a single organism [4]. Contamination was defined as growth of three or more organisms.

Data collection

Demographic and obstetric data (age, trimester, parity, gravida, religion, urban/rural residence, and symptoms) were recorded using a structured proforma. ASB was diagnosed when a patient had no urinary symptoms (dysuria, frequency, urgency, suprapubic pain, fever) but had a positive culture [4].

Statistical analysis

Data were entered into Microsoft Excel (Microsoft Corporation, Redmond, WA) and analyzed using Statistical Package for the Social Sciences version 25.0 (IBM Corp., Armonk, NY). Categorical variables were expressed as frequencies and percentages. Comparisons between groups were performed using the chi‑square test. A p value <0.05 was considered statistically significant. For all tables, the chi‑square value (χ²) and the corresponding p value are reported.

## Results

A total of 310 pregnant women were screened. Their mean age was 26.4 years (range 18-39 years). The overall culture positivity rate was 90 of 310 (29.0%). Among the 90 culture‑positive women, 65 (72.2%) had no urinary symptoms (ASB), while 25 (27.8%) were symptomatic.

Table 2 shows the age distribution of clinically suspected UTI. The highest positivity was observed in the 25-29-year age group (36/117, 30.8%), followed by the 20-24-year age group (34/118, 28.8%). The difference across age groups was statistically significant (χ² = 4.884 (df = 4), p = 0.027).

**Table 2:** Age distribution of pregnant women clinically suspected of UTI (n = 310) ^*^Chi-square test for trend across age groups UTI: urinary tract infection

Age range (years)	Number of patients	Culture positivity, n (%)	χ² value	p value^*^
<19	37	7 (18.9%)	4.884	0.027
20-24	118	34 (28.8%)		
25-29	117	36 (30.8%)		
30-34	48	11 (22.9%)		
35-39	4	2 (50.0%)		
Total	310	90 (29.0%)		

Table 3 presents the trimester‑wise distribution of culture positivity. The third trimester had the highest number of positive cases (50/147, 34.0%), followed by the second trimester (21/94, 22.3%) and the first trimester (19/69, 27.5%). The association between trimester and culture positivity was statistically significant (χ² = 8.896 (df = 2), p = 0.012).

**Table 3:** Distribution of urinary tract infection in pregnant women by trimester (n = 310) ^*^Chi-square test (two degrees of freedom)

Trimester	Number of patients	Culture positivity, n (%)	χ² value	p value^*^
First	69	19 (27.5%)	8.896	0.012
Second	94	21 (22.3%)		
Third	147	50 (34.0%)		
Total	310	90 (29.0%)		

Table 4 combines the distribution by parity and gravida. For parity, primigravida women (parity 0) had the highest number of positive cases (43/142, 30.3%), followed by women with one previous delivery (parity 1: 34/131, 26.0%). The association was statistically significant (χ² = 7.192 (df = 2), p = 0.027). For gravida, Gravida 1 women contributed 41 of 132 (31.1%) positive cases, and Gravida 2 contributed 36 of 138 (26.1%). The association was highly significant (χ² = 12.458 (df = 2), p = 0.002).

**Table 4:** Distribution of urinary tract infection in pregnant women by parity and gravida (n = 310) ^*^For parity: chi-square test for parity 0 vs. ≥1. For gravida: chi-square test for gravida 1-2 vs. ≥3

Variable	Category	Number of patients	Culture positivity, n (%)	χ² value	p value^*^
Parity	0	142	43 (30.3%)	7.192	0.027
	1	131	34 (26.0%)		
	2	33	11 (33.3%)		
	3	3	2 (66.7%)		
	4	1	0 (0.0%)		
Gravida	1	132	41 (31.1%)	12.458	0.002
	2	138	36 (26.1%)		
	3	36	11 (30.6%)		
	4	3	2 (66.7%)		
	5	1	0 (0.0%)		

Table 5 shows that, among the 25 symptomatic culture‑positive women, urgency was the most common symptom (12/25, 48.0%), followed by abdominal pain (9/25, 36.0%) and burning micturition (7/25, 28.0%).

**Table 5:** Distribution of urinary tract infection in pregnant women by symptoms among symptomatic positive cases

Symptoms (among symptomatic positive cases, n = 25)	Number of patients (%)
Urgency	12 (48.0%)
Abdominal pain	9 (36.0%)
Burning micturition	7 (28.0%)
Bleeding	6 (24.0%)

## Discussion

The present study found a culture positivity rate of 90 of 310 (29.0%) among pregnant women attending a tertiary care hospital in Jaipur. Of these, 65 of 90 (72.2%) were asymptomatic, confirming that the vast majority of UTIs in pregnancy are clinically silent. This proportion is comparable to the 74.8% reported by Rizvi et al. from Aligarh [11] and the 83.3% reported by Sonkar et al. from Lucknow [12], but higher than the 58.9% reported by Patil et al. from Nagpur [9]. The high rate of ASB underscores the inadequacy of relying on symptoms alone for diagnosis.

The highest culture positivity was observed in the third trimester (50/147, 34.0%). This is consistent with several previous studies. Sibi et al. reported that 57.2% of UTIs occurred in the third trimester [13], and Eshwarappa et al. found 68.1% [14]. The physiological changes of late pregnancy, including maximal ureteral dilatation, urinary stasis, and increased vesicoureteral reflux, likely explain this peak [2,3].

Women aged 25-29 years had the highest positivity rate (36/117, 30.8%). This matches the findings of Kant et al. (25-30 years) [15] and Sujatha and Nawani (21-30 years) [16]. This age range represents the peak reproductive period, with higher sexual activity and greater likelihood of prior UTI episodes [1].

Primigravida and gravida 1 women accounted for nearly half of all positive cases (43/90, 47.8% for parity 0; 41/90, 45.6% for gravida 1). This finding is similar to the 54.2% primigravida prevalence reported by Patil et al. [9] but contrasts with the 60% multigravida predominance reported by Ngong et al. [17]. The higher susceptibility in first pregnancies may be related to lack of prior immune adaptation to uropathogens or differences in hygiene practices.

Urban residence was associated with a higher number of positive cases (54/90, 60.0%), but this was not statistically significant (p = 0.096). The lack of significance may be due to the large proportion of urban women in the study population (219/310, 70.6%). Belete and Saravanan also reported a higher urban prevalence (92%) [10], while Sonkar et al. found a rural predominance (70.8%) [12], suggesting geographic variability.

The most common symptom among the minority of symptomatic women was urgency (12/25, 48.0%), followed by abdominal pain (9/25, 36.0%). This is similar to the findings of Johnson et al., who reported urgency in 59.2% of symptomatic pregnant women [18].

Limitations

This study has several limitations. First, it was conducted at a single tertiary care center, which may limit generalizability to primary and secondary health facilities in rural areas. Second, the study period was relatively short (five months), and seasonal variations in UTI prevalence could not be assessed. Third, we did not follow the patients longitudinally to determine progression from ASB to symptomatic infection or adverse pregnancy outcomes. Fourth, antibiotic use before sample collection was self‑reported, which may introduce recall bias.

## Conclusions

ASB affects nearly three‑quarters of pregnant women with culture‑positive UTI in this tertiary care setting. The highest prevalence is observed in the third trimester and among primigravida women. Routine urine culture screening for all pregnant women, regardless of symptoms, is essential to detect silent infections and prevent progression to pyelonephritis and adverse pregnancy outcomes. Future studies should focus on the cost‑effectiveness of universal screening in primary care settings and on interventions to improve antibiotic stewardship in pregnancy.
